# Synthesis of Radioluminescent CaF_2_:Ln Core, Mesoporous Silica Shell Nanoparticles for Use in X-ray Based Theranostics

**DOI:** 10.3390/nano10081447

**Published:** 2020-07-24

**Authors:** Hayden Winter, Megan J. Neufeld, Lydia Makotamo, Conroy Sun, Andrea M. Goforth

**Affiliations:** 1 Department of Chemistry, Portland State University, 1719 SW 10th Ave., Portland, OR 97201, USA; hwinter@pdx.edu (H.W.); lym2@pdx.edu (L.M.); 2 Department of Pharmaceutical Sciences, College of Pharmacy, Oregon State University, Portland, OR 97201, USA; neufeldm@oregonstate.edu; 3 Department of Radiation Medicine, 3181 S. W. Sam Jackson Park Rd, Oregon Health & Science University, Portland, OR 97239, USA

**Keywords:** CaF_2_, mesoporous silica, nanoparticle, core-shell, lanthanide-doped, polyethylene glycol, hydrothermal annealing, radioluminescence, radiotherapy

## Abstract

X-ray radiotherapy is a common method of treating cancerous tumors or other malignant lesions. The side effects of this treatment, however, can be deleterious to patient quality of life if critical tissues are affected. To potentially lower the effective doses of radiation and negative side-effects, new classes of nanoparticles are being developed to enhance reactive oxygen species production during irradiation. This report presents the synthesis and radiotherapeutic efficacy evaluation of a new nanoparticle formulation designed for this purpose, composed of a CaF_2_ core, mesoporous silica shell, and polyethylene glycol coating. The construct was additionally doped with Tb and Eu during the CaF_2_ core synthesis to prepare nanoparticles (NPs) with X-ray luminescent properties for potential application in fluorescence imaging. The mesoporous silica shell was added to provide the opportunity for small molecule loading, and the polyethylene glycol coating was added to impart aqueous solubility and biocompatibility. The potential of these nanomaterials to act as radiosensitizers for enhancing X-ray radiotherapy was supported by reactive oxygen species generation assays. Further, in vitro experiments indicate biocompatibility and enhanced cellular damage during X-ray radiotherapy.

## 1. Introduction

X-ray radiation plays a key role in various diagnostic and treatment modalities in modern medicine. In part, the high photon energy of X-rays allows for greater tissue penetration depth compared to lower energy photons. As such, new diagnosis and treatment modalities are being developed using X-ray responsive materials [1,2,3]. Upon irradiation, radiation responsive materials absorb energy from X-rays through various interactions, which results in the production of lower energy photons in secondary processes that can subsequently cause specific chemical reactions or radioluminescence (RL) [4,5,6]. The observation of these material-specific X-ray activated characteristics has resulted in numerous studies into the synthesis and application of materials for cancer-based theranostics.

X-ray radiotherapy (XRT) is a common treatment modality for cancerous tumors which is often delivered in the form of external beam radiation. In addition to direct cellular damage, radiation therapy works indirectly by generating chemically reactive small molecules and molecular radicals, largely through radiolysis of water. These reactive species subsequently react with critical cellular components (e.g., DNA), thus decreasing survival and cellular replication. However, as XRT damages everything within the path of the radiation, including healthy tissue, radiosensitizers have been studied as a means of selectively multiplying local cellular damage in cancerous tissues during XRT [2].

Inorganic nanoparticles (NPs) have shown promise as radiosensitizers due to their high structural density and composition variability. Compared to bodily tissues, denser, higher atomic number nanomaterials have higher probability interactions with X-rays, and thus better X-ray attenuation is expected. These interactions include X-ray absorption and scattering by inorganic NPs, accompanied by deposition of energy from the incident photons within the local microenvironment surrounding the NPs [4,5]. A critical consequence of this energy deposition is increased generation of reactive oxygen species (ROS), as well as a variety of additional chemically reactive species (e.g., molecular radicals), leading to damage of the surrounding cellular structures [7]. The capability of NPs for this type of enhancement was initially demonstrated by Hainfeld et al. in 2004, using Au NPs to sensitize XRT [8]. Whether relying on physical enhancement by dense materials that strongly attenuate X-rays, or directing energy from X-ray photons to specific chemical pathways, inorganic NPs have widely been investigated as radiotherapeutic agents [8,9,10,11,12,13].

Alongside radiotherapeutic strategies, some inorganic NPs have also been designed for use in X-ray activated diagnostic imaging. Inorganic NPs that can convert absorbed energy from incident X-rays into visible photons can enable radioluminescence (RL) imaging, by locating the NPs with high spatial resolution via their visible-range photon emission [6,14]. Our group and several others have realized this application in vivo, for example with the use of Ba_0.55_Y_0.3_F_2_:Eu^3+^ by our group or Eu-doped NaGdF_4_ NPs by Sudheendra et al. [15,16]. In these cases, luminescent europium ion lattice dopants in the nanocrystals exhibited characteristic strong emissions in the visible spectrum, between 500–700 nm. This wavelength range corresponds well with effective tissue transmission [17]. These properties, along with their strong X-ray interactions, illustrate the potential of luminescent NPs such as these to act as both diagnostic and therapeutic agents.

Calcium fluoride (CaF_2_) is a popular optical matrix for designing luminescent materials and has additional properties that make it useful for RL applications, such as RL-based biomedical imaging. The photonic and thermal properties of CaF_2_, such as its large bandgap (12 eV) and low phonon energy, reduces non-radiative energy loss and increases the probability of radiative decay at the dopant site, making CaF_2_ an effective host matrix in the visible to UV range. Further, the similar ionic radius of Ca^2+^ to various fluorescent lanthanide ions, as well as the high symmetry of the cation sites in the face-centered cubic structure of CaF_2_, makes the inclusion of such dopants straightforward and permits the symmetries of their excited states [18,19]. Additionally, CaF_2_ has a density of 3.18 g/cm^3^ and a higher average atomic number than most biological tissues, properties that increase the probability of X-ray interactions. Correspondingly, a variety of syntheses have been developed to formulate doped CaF_2_ NPs for photonics-based applications, including those in the biomedical field [20,21,22,23]. Typical syntheses of CaF_2_ NPs are performed using coprecipitation procedures in either water or short-chain alcohols [24,25,26,27]. However, while the large lattice enthalpy of CaF_2_ makes coprecipitation highly thermodynamically accessible, the relatively fast reaction rate limits overall reaction control.

In this work, CaF_2_ and Ln-doped CaF_2_ NPs (Ln = Eu or Tb) were synthesized using an aqueous coprecipitation method followed by hydrothermal annealing. This method capitalizes on the synthetic ease of coprecipitation methods, ease of lanthanide ion doping, and the ability to access larger single-crystal NP sizes through hydrothermal annealing [27,28]. While many syntheses of CaF_2_ NPs employ a hydrothermal annealing step, this work correlates the effect of annealing over time to changes in the Ln-doped CaF_2_ NPs crystallite size and luminescence emission intensity, intending to increase radioluminescence quantum yield. Results suggest that the fusion of grain boundaries occurs rapidly, improving quantum yield, while the annealing of defect sites within the crystallites occurs at a slower rate and is also critical for improving the quantum yield. After improving the luminescence of the NPs, they were then coated with a mesoporous silica (MS) shell, followed by covalently bound polyethylene glycol-silane (PEG-silane). The NP products are thoroughly characterized through the synthetic steps leading to the overall CaF_2_:Ln-MS-PEG NP constructs, using transmission electron microscopy (TEM), X-ray diffraction (XRD), energy-dispersive X-ray spectroscopy (EDX), Fourier transform infrared spectroscopy (FT-IR), dynamic light scattering (DLS), and radioluminescence (RL).

Further, we demonstrate in vitro that the strongly radioluminescent MS:Ln-CaF_2_ NPs are highly biocompatible in the absence of X-rays, while decreasing the viability of CT26 colorectal cancer cells under XRT conditions. Thus, the novel CaF_2_:Ln-MS-NP constructs synthesized and reported herein are promising for further study as a combined radiotherapeutic and radiodiagnostic in XRT and RL-guided spatial imaging applications.

## 2. Materials and Methods

### 2.1. Materials

The following chemicals were purchased and used without purification: For CaF_2_ synthesis: CaCl_2_ (99%, anhydrous, Acros, Geel, Belgium), TbCl_3_·6H_2_O (99%, Acros, Geel, Belgium), EuCl_3_·6H_2_O (99%, Sigma Aldrich, St. Louis, MO, USA), Sodium citrate dihydrate (99%, J.T. Baker, Phillipsburg, NJ, USA), Ammonium fluoride (98%, Sigma-Aldrich, St. Louis, MO, USA), ethanol (95%, Thermo-Fisher, Waltham, MA, USA). For Mesoporous silica coating: Cetyltrimethyl ammonium bromide (99%, Research Organics, Cleveland, OH, USA), ethanol (200 proof, Decon Labs, King of Prussia, PA, USA), tetraethoxysilane (98%, Sigma-Aldrich, St. Louis, MO, USA), triethylamine (99%, Acros, Geel, Belgium), 2-[methoxy(polyethyleneoxy)9-12propyl]trimethoxysilane (tech grade, 70+%, Gelest, Morrisville, PA, USA). For ROS quantification assays: aminophenyl fluorescein (APF, 99%, Thermo-Fisher, Waltham, MA, USA), Singlet Oxygen Sensor Green (SOSG, 99%, Thermo-fisher, Waltham, MA, USA) (All reagents in all experiments used as received).

### 2.2. CaF_2_ and Ln-Doped CaF_2_ Nanoparticle Syntheses

**Scheme 1:**
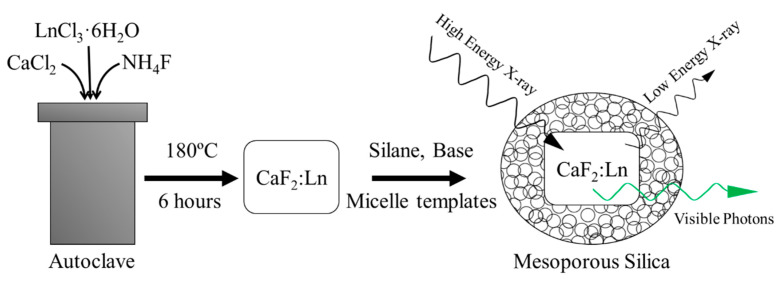
Schematic outline of the synthesis of CaF_2_:Ln-MS NPs used in this work and their X-ray active properties.

Scheme 1 outlines the following procedures including CaF_2_ NP synthesis and mesoporous silica coating. In a typical synthesis, a 50 mL round bottom flask was loaded with calcium chloride, or calcium and lanthanide chlorides (Tb or Eu). The reagent masses used are detailed in Table 1 and were chosen to target nanomaterials with ~20 atomic percent Ln dopant in place of Ca ions.

Along with the metal chlorides, 5.17 g of sodium citrate dihydrate was added, followed by 22 mL of distilled water. This solution was stirred for 10 min at room temperature (25 °C) to allow the solids to dissolve completely. The resulting concentrations in solution were then 126.14 mM CaCl_2_, 9.96 mM LnCl_3_·6H_2_O, and 0.7 M sodium citrate dihydrate. For undoped CaF_2_ NPs, the CaCl_2_ concentration was 139.84 mM. Meanwhile, 322 mg of ammonium fluoride was dissolved in 5 mL of distilled water (1.74 M) in a 15 mL centrifuge tube. This solution was loaded into a burette. Then, while still under continuous stirring, ammonium fluoride solution was added dropwise into the round bottom flask containing the metal chloride solution, at a rate of approximately one drop per second. After all the fluoride solution was added, the slightly turbid solution in the round bottom flask was divided evenly into three 20 mL Teflon autoclave liners, which were placed in stainless steel autoclaves (Parr, Moline, IL, USA) and rapidly heated to a temperature of 180 °C for 6 h. Following heating, the autoclaves were subsequently cooled to room temperature, and the product solutions in the three autoclaves were collected together into a 50 mL centrifuge tube. Then, 10 mL of ethanol (95%) was added, and the product suspensions were centrifuged to isolate the NPs. The NPs were again redispersed in a solution of 5 mL of water and 35 mL of ethanol, followed by centrifugation. This process was repeated until the dark carbonized species left over from the reaction were no longer present. Finally, the CaF_2_ (or CaF_2_:Ln) NPs were dried overnight at 50 °C, then crushed to a fine powder using a mortar and pestle and stored for further use.

### 2.3. Mesoporous Silica Coating

Into a 250 mL round bottom flask were added 54 mg of dried, powdered CaF_2_ NPs, and 100 mL of distilled water, followed by sonication for 30 min. Then, 10 mL of ethanol (200 proof), 84 µL of triethylamine (to attain a concentration of 5.48 µM), and 1.8 g of cetyltrimethyl ammonium bromide (CTAB) (44.9 mM) were added to the flask. The reaction mixture was next heated to 80 °C and stirred at 1400 rpm with a 3 cm almond-shaped stir bar, which resulted in the formation of CTAB micelles. Next, 300 µL of tetraethoxysilane (TEOS) (12.3 mM) was added to the flask dropwise over 20 s. The flask was subsequently heated and stirred for an additional 30 min to accomplish the hydrolysis and polycondensation of the TEOS around the CTAB, followed by cooling in a stirring water bath for 60 s. The reaction products were then collected in centrifuge tubes, followed by centrifugation to isolate the silica-coated CaF_2_ NP products. The silica-coated CaF_2_ NPs were next resuspended in 20 mL of ethanol (200 proof), followed by 10 min of sonication. This colloidal suspension was subsequently transferred to a 100 mL round bottom flask, and then 2 mL of 12 M hydrochloric acid was added to remove the CTAB within the silica network and render the silica coating mesoporous (MS = mesoporous SiO_2_). The resulting colloidal suspension containing the MS-CaF_2_ NPs was stirred and heated to 80 °C for 30 min. Then it was cooled in a stirring water bath, and solids were collected by centrifugation. The isolated MS-CaF_2_ NPs were purified two cycles of redispersion in a 1 mL water and 20 mL ethanol solution, followed by centrifugation. Finally, the MS-CaF_2_ NPs were dried at 50 °C, milled to a powder in a mortar and pestle, and stored for further use.

### 2.4. Surface Modification by Covalent Attachment of Polyethylene Glycol

15 mg of MS-CaF_2_ NPs were suspended in 20 mL of distilled water in a 100 mL round bottom flask, followed by sonication. While stirring at room temperature, 40 µL of ammonium hydroxide (30% *w/w* in water) was added, followed by 100 µL of 2-[methoxy(polyethyleneoxy)9-12propyl]trimethoxysilane (PEG-silane). After PEG-silane addition, the reaction mixture was left stirring at room temperature for two hours. At the end of this time, the reaction products were poured into a centrifuge tube, and 10 mL of ethanol was added to flocculate the pegylated MS-CaF_2_ NPs (PEG-MS-CaF_2_ NPs). The PEG-MS-CaF_2_ NPs were collected by centrifugation, followed by resuspension in 10 mL of 1:1 water: ethanol and centrifuging, and additional purification by 2 more cycles of redispersion and centrifuging with 10 mL of ethanol. Finally, the PEG-MS-CaF_2_ NP products were dried overnight at 50 °C, then crushed to a fine powder using a mortar and pestle, and stored for further use.

### 2.5. Instrumental Analysis

The NP products through the stages of synthesis were characterized using a combination of transmission electron microscopy (TEM), energy dispersive X-ray spectroscopy (EDX), X-ray diffraction (XRD), radioluminescent spectroscopy, and x-ray photoelectric spectroscopy (XPS).

Transmission Electron Microscopy was performed on a Tecnai F20 TEM (FEI, Hillsboro, OR, USA) operating at 4200 eV. Samples were prepared by dispersing NPs in ethanol (ACS) at concentrations of approximately 1 mg/mL and drop-casting a single drop onto type-B carbon-coated copper TEM grids (Ted Pella Product #1844-F, Redding, CA, USA). These samples were dried at room temperature for 10 min before drying in a 50 °C oven for at least one hour before imaging. The mean diameters of the nanoparticle samples were determined by analyzing several TEM images, corresponding to different (representative) areas of each sample grid. ImageJ software (version 1.51n, National Institutes of Health, Bethesda, MD, USA) was used to measure the NPs and produce size distributions; each reported size value is based on the minimum requirement to measure at least 200 NPs. To prevent measurement bias, the minimum criteria for an individual image used in mean diameter determination were that it must include at least 50 NPs and that all NPs in the image had to be measured. EDX measurements were taken using the same instrument in scanning-TEM mode and collected using an Oxford Instruments EDX detector (Abingdon, Oxfordshire, England).

X-ray diffraction patterns were collected using a Rigaku Ultima IV X-ray diffraction system (Tokyo, Japan) using graphite monochromatized Cu Kɑ radiation. Samples were prepared by grinding dry powders in a mortar and pestle and placing them in a glass sample holder. Patterns were collected with a continuous movement measurement at a resolution of 0.05° rate of 1 degree per minute from 20 to 60° 2θ. The resulting data was analyzed using Rigaku PDXL2 software. Crystallite size was estimated using the Scherrer equation (Equation (1)) in which τ is the mean crystallite size, K is the shape factor which is assumed as a sphere, λ is the X-ray wavelength, β is the peak width at half of the peak maximum, and θ is the X-ray angle.

(1) $$\tau =\frac{K\lambda }{\beta \cos\theta }$$

FT-IR spectra were obtained using a Thermo Scientific Nicolet iS10 spectrometer (Waltham, MA, USA) fitted with an attenuated total reflectance attachment having a diamond window. Solid samples were first dried of solvents by heating at 125 °C, then placed directly on the diamond window and compressed. Spectra were averaged from 32 scans, taken at a 1 cm^−1^ resolution between 650–4000 cm^−1^.

For the Ln-doped, CaF_2_ NPs, photoluminescence (PL) spectroscopy was performed using a Shimadzu RF-5301PC fluorimeter (Canby, OR, USA). The Ln-doped, CaF_2_ NPs colloidal solutions used in these measurements were prepared by sonicating the corresponding dry powders in water at a concentration of 1 mg/mL. X-ray excited luminescence spectra were also acquired, using 30 mg of dry Ln-doped CaF_2_ NP powder for each measurement, and a CellRad Faxitron X-ray system (Precision X-ray Irradiation, North Branford, CT, USA) operating at 130 kV and 5 mA as the excitation source; no beam filter was applied. The X-ray excited luminescence was recorded using a StellarNet Silver Nova 200 spectrometer (Tampa, FL, USA) attached to a collimating lens via armored fiber-optic cable (Thorlabs, Newton, NJ, USA)). These measurements were collected with an integration time of 30 s.

X-ray Photoelectron Spectroscopy (XPS) was performed on a Versaprobe II XPS/AES (ULVAC-PHI, Chanhassen, MN, USA) featuring an aluminum X-ray source. Samples were prepared for XPS measurements by drop-casting NP suspensions in methanol onto individual aluminum foil substrates heated at 50 °C, followed by drying the samples under vacuum at room temperature for 12 h before placement in the XPS transfer arm. Once in the vacuum chamber, the samples were bombarded with X-rays from the source, in circular spots with diameters of 100 µm. The electron beam used in these experiments was operating at 125 V and 50 W, and charge balancing was provided by both an e-neutralizer and argon-derived ion beam. The spectra were calibrated to the carbon 1s peak present in each spectrum.

Dynamic light scattering (DLS) was performed using a Malvern Nano ZSP (Malvern Panalytical, Malvern, UK) to determine the size, zeta potential, and polydispersity. Samples were prepared by dispersing 100 µg of NPs in 1 mL of millipure water, briefly sonicating, then transferring to a DLS cuvette with gold-plated contacts for zeta potential measurements.

### 2.6. ROS Quantification Assays

Stock colloidal solutions of CaF_2_:Tb-MS-PEG were prepared in water, with fixed concentrations of 1 µg NP per 1 mL of water. Each colloidal solution was sonicated for at least 5 min before it was plated in the wells of a 96-well plate, each well containing 100 uL of the NP stocks. Some wells contained only millipure water instead of nanoparticle solutions as a control. Next, either 10 uL of a 10 μM APF solution or 10 uL 10 μM SOSG solution were added to the sample and control wells. These plates were transferred to a Faxitron Cellrad X-ray irradiator (Precision X-ray Irradiation, North Branford, CT, USA) and irradiated at 130 kVp and 20 mA for variable amounts of time to attain different delivered radiation doses (2,4,6,8,10,12,14 Gy). As the pre-determined dosing conditions were met, triplicates of wells were removed from the irradiator to a separate 96 well plate in which the solutions would be analyzed. After all conditions were collected, the 96 well plates were analyzed for photoluminescence using an Infinite M200 Pro plate reader (Tecan US Inc, Morrisville, NC, USA.) APF fluorescence was measured using an excitation wavelength of 490 nm, while monitoring emission intensity at 520 nm, and SOSG fluorescence was measured using an excitation wavelength of 500 nm, while monitoring emission intensity at 525 nm.

### 2.7. In Vitro Cell Viability

Murine fibroblast (NIH 3T3) and human liver carcinoma (HepG2) cells (both sourced from ATCC, Manassas, VA, USA) were cultured in appropriate growth media and seeded at a density of approximately 10,000 cells per well into a 96-well plate. Cells were allowed to settle and grow for approximately 24 h prior to dosing, with either media containing CaF_2_:Tb-MS-PEG NPs, or a control solution. A dosing plate was prepared at a maximum concentration of 500 µg/mL for CaF_2_:Tb-MS-PEG in media. The maximum concentration was then serially diluted by half to prepare lower concentrations (500, 250, 125, 62, 31, 15, 7.5, 3.7, and 1.9 µg/mL). All dilutions were added to plates in triplicate (technical replicates) at 100 µL per well for 24 h. Following exposure of the cells to the NP solutions (or blank media), cell viability was assayed using alamarBlue^TM^ cell viability reagent (10% vol/vol in media, Thermo-Fisher, Waltham, MA, USA) where the wells were individually irradiated using an excitation wavelength of 530 nm and emission was monitored at 590 nm with an Infinite M200 Pro plate reader (Tecan US Inc, Morrisville, NC, USA). All results were normalized to untreated cells. All experiments were performed in triplicate (biological replicates).

### 2.8. In Vitro XRT

Murine colorectal cancer (CT26) cells (ATCC, Manassas, VA, USA) were grown in the appropriate media and seeded at a density of approximately 2000 cells in a 96 well plate. Cells were allowed to settle and grow for approximately 24 h prior to dosing. A dosing plate was prepared at a maximum concentration of 500 µg/mL for CaF_2_:Tb-MS-PEG in media. The maximum concentration was then serially diluted by half to prepare lower concentrations (500, 250, 125, 62, 31, 15, 7.5, 3.7, and 1.9 µg/mL). Cells were then incubated with the CaF_2_:Tb-MS-PEG NPs for 24 h, then the media was removed and replaced prior to 4 Gy radiation at 130 kVp using a Faxitron CellRad irradiator (Precision X-ray Irradiation, North Branford, CT, USA). Cells were than incubated for an additional 24 h and cell viability was measured using alamarBlue^TM^ cell viability reagent (Thermo-Fisher, Waltham, MA, USA) and measured on an Infinite M200 Pro plate reader (Tecan US Inc, Morrisville, NC, USA) at 530 nm excitation and 590 nm emission. All results were normalized to untreated cells. All experiments were performed in triplicate (biological replicates).

## 3. Results and Discussion

### 3.1. Synthesis and Impacts of Hydrothermal Annealing on Crystallite Size

**Figure 1:**
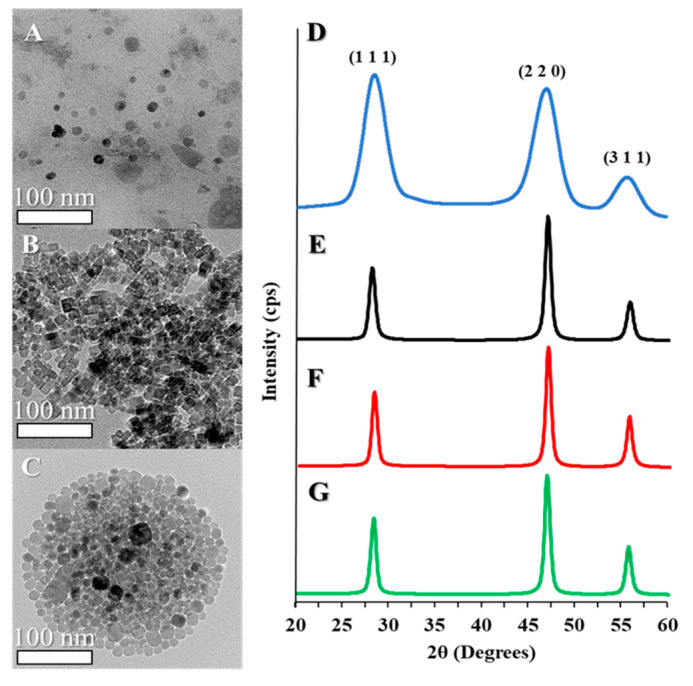
Transmission electron microscopy (TEM) images of: (**A**) CaF_2_ nanoparticles (NPs) before and (**B**) after hydrothermal annealing, and (**C**) Tb-doped CaF_2_ NPs after hydrothermal annealing. X-ray diffraction (XRD) patterns of CaF_2_ NPs (**D**) before and (**E**) after hydrothermal annealing, and (**F**) Eu-doped and (**G**) Tb-doped CaF_2_ NPs.

Synthesis of CaF_2_ NPs was achieved by a modified method adapted from Pedroni et al., who developed a synthesis to study near-IR to visible photon conversion in NP optical matrices [21]. In this procedure, an aqueous solution containing calcium ions, or calcium and lanthanide ions, is introduced to a separate solution of fluoride ions within an environment highly concentrated with citrate. The citrate acts as a steric and electrostatic surface stabilizer. Due to the large lattice enthalpy that causes calcium and lanthanide ions to precipitate in the presence of fluoride, CaF_2_ NPs or Ln-doped CaF_2_ NPs readily form upon mixing the component ion solutions. The as-prepared NPs are typically pseudo-spherical and have diameters ranging from 10–20 nm (Figure 1A). Subsequently, the as-prepared NPs were annealed in a hydrothermal cell for 6 h at 180 °C, which resulted in the formation of highly regular cubic nanocrystals; the overall mean diameter for a representative CaF_2_ NP sample following annealing is 13.6 ± 3.5 nm (Figure 1B), still within the typical range of sizes prior to hydrothermal treatment. Estimates of the elemental composition of the CaF_2_ NPs via EDX measurements showed particle compositions close to the expected ratio of 1:2 calcium: fluorine in the undoped CaF_2_ NPs (Figure S1). In comparison, when 15% of the calcium ions were replaced with either terbium or europium ions in the metal ion precursor solution, EDX measurements showed that the Ln ion accounted for approximately 18% of the cations in the resulting CaF_2_:Ln NPs (Figure S1). By TEM, it was observed that the ~18% replacement of calcium ions with either terbium or europium ions did not significantly alter the typical diameters or morphologies of NPs; for example, a typical sample of CaF_2_:Tb NPs after hydrothermal annealing (Figure 1C, Table S1) has a mean diameter of 13.4 ± 4.2 nm, and is still somewhat faceted.

While hydrothermal annealing did not substantively alter the TEM-observable mean diameters of the NPs, changes in the crystallite size were observable by PXRD. PXRD patterns before and after hydrothermal treatment (Figure 1D,E) both reveal the expected diffraction pattern of the fluorite-type CaF_2_ lattice; all peaks can be indexed to fluorite CaF_2_ (PDF Card 35816), and there are no obvious crystalline impurities. Noticeable peak broadening is observed both before and after hydrothermal treatment, and the FWHM of the peaks can be used to estimate the crystallite size using the Scherrer equation. Before hydrothermal annealing, the NPs have a calculated mean diameter of 3.2 ± 1.8 nm, which increases to 10.2 ± 1.6 nm following annealing. The similarity between the PXRD-determined crystallite size and the TEM-measured diameter of the NPs post-annealing indicates that the annealed NPs are single crystalline, whereas they were likely polycrystalline prior to the treatment [29,30]. It is, therefore, reasonable to conclude that the annealing treatment reduces grain boundaries within the as-prepared NPs, more so than it results in the ripening of the as-prepared NPs into larger particles.

Finally, in comparison of the PXRD patterns of Ln-doped vs. undoped CaF_2_ NPs following annealing, it was observed that both the peak positions and the crystallite sizes are essentially the same at the ~18% level of Ln doping (Figure 1F,G); that is, the addition of Ln-dopant does not result in substantive structural or size changes in the product NPs.

### 3.2. Influence of Crystallite Size on Lanthanide Ion Photoluminescence Intensity

**Figure 2:**
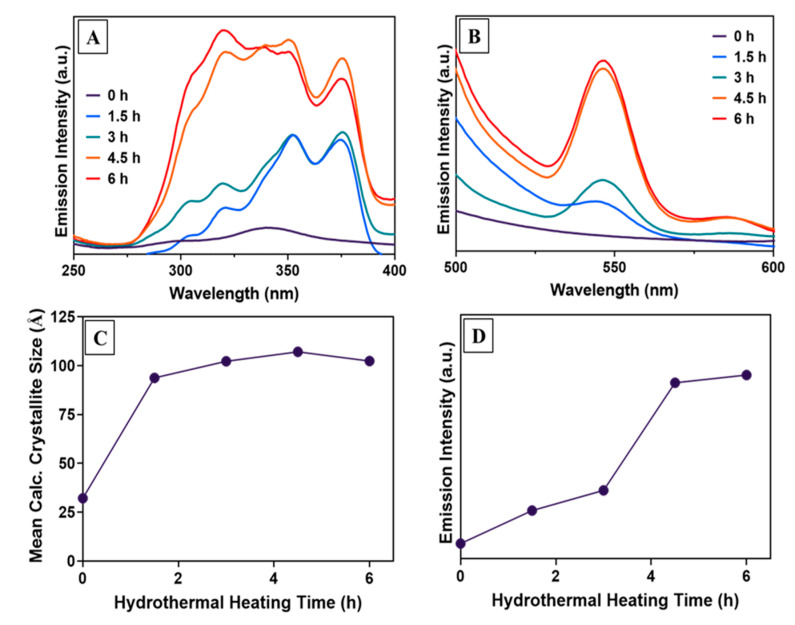
(**A**) Excitation spectrum of CaF_2_:Tb NPs with variable hydrothermal annealing times, monitoring the intensity of the 545 nm emission. (**B**) Emission intensity of the samples when excited by 320 nm radiation. (**C**) Crystallite size data estimated from XRD patterns of each sample using the Scherrer equation. (**D**) Emission intensity at 545 nm plotted vs. increasing hydrothermal heating time.

The effect of the observed increase in crystallite size with annealing on Ln ion luminescence intensity was next investigated. Hypothesizing that increases in crystallite size would correlate with increased lanthanide ion emission intensity, photoluminescence excitation and emission spectra were monitored over time by removing aliquots of Tb:CaF_2_ NPs at different time points during the hydrothermal treatment For the Tb-doped CaF_2_ NPs, changes to the photoluminescent properties were observed by monitoring the Tb ^5^D_4_ → ^7^F_5_ transition at 545 nm (Figure 2A,B); corresponding PXRD measurements were also performed on these aliquots, and crystallite size was estimated from line broadening (Figure 2C).

The as-prepared CaF_2_:Tb NPs were observed to be only weakly photoluminescent, having low initial absorptivity across the 300–400 nm range, and negligible emission intensity in the visible region. With increased annealing time, absorptivity initially increased over the wavelength range 300–400 nm. At longer time points, increased absorptivity extended down to 275 nm (Figure 2A). These increases in absorptivity were well correlated with increased photoluminescence intensity in the visible region. For example, the increase in intensity at 545 nm, which was initially significant, is even more dramatic at longer annealing times as the absorptivity at shorter wavelengths increases (Figure 2B). Both the excitation and emission spectral changes plateau after 4.5 h.

While the photoelectronic properties are observed to plateau only after 4.5 h, by PXRD line broadening it was observed that the mean crystallite size no longer increased after 1.5 h of hydrothermal treatment (Figure 2C); that is, the photoluminescence emission intensity continued to increase while the crystallite size did not (Figure 2D). The further increase in emission intensity with hydrothermal heating time (up to 6 h) is most likely the result of the reduction in the concentrations of point defects within the CaF_2_ matrices, leading to decreasing probability of non-radiative decay of the nanocrystal excited state at those sites and increasing probability of radiative decay at Ln-ion dopant sites [31,32].

### 3.3. Mesoporous Silica Coating and Organosilane Surface Modification

**Figure 3:**
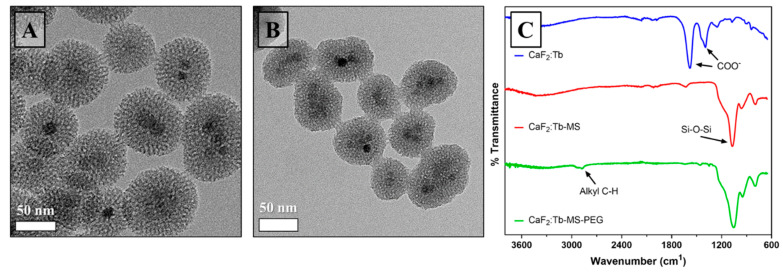
Surface modification of CaF_2_:Tb NPs. TEM images of (**A**) CaF_2_:Tb-MS NPs and (**B**) CaF_2_:Tb-MS-PEG NPs. (**C**) Fourier transform infrared spectroscopy (FT-IR) spectra of as-prepared CaF_2_:Tb NPs (top), CaF_2_:Tb-MS NPs (middle), and CaF_2_:Tb-MS-PEG NPs (bottom).

Following successful synthesis of citrate-stabilized CaF_2_:Ln NPs, a mesoporous silica (MS) coating was subsequently deposited on the NP surfaces to prepare CaF_2_:Ln-MS NPs (Figure 3A). This MS coating was applied for several reasons. First, the as-prepared citrate-stabilized NPs were not easily redispersed as stable aqueous colloids, thus limiting their biological potential; we hypothesized that the MS coating would increase the hydrophilicity of the NPs to promote colloidal stability [33]. Further, the MS coating provides the opportunity to covalently modify the NP surfaces with a wide variety of commercially available organosilane reagents [34], thus permitting further synthetic alterations to increase colloidal stability and biological utility. Finally, MS NPs on their own are popular biomedical formulations due not only to their hydrophilicity, but also to their ability to carry and release an encapsulated small molecule payload. Mesoporous silica NPs have pores with sizes that are tailorable in synthesis, and as such, they have been studied as host vehicles in a variety of drug delivery, therapeutic, and imaging applications as well as non-medical uses such as food active packaging [9,35,36,37].

To prepare CaF_2_:Ln-MS NPs, we modified a literature procedure used in the production of hollow mesoporous silica NPs, where relatively thin mesoporous silica shells were uniformly deposited onto solid silica cores that were later removed by selective structural etching [38]. Following deposition of the MS coating, morphological evaluation of a sample of CaF_2_:Tb-MS NPs by TEM revealed that occurrences of multiple CaF_2_ cores in single MS shells were commonly observed, and that isolated particles had an overall average diameter of 55 ± 9 nm (Figure 3). Since the overall average diameter is less than 100 nm, these NP constructs are potentially appropriately sized for use as intravenously administered agents [39,40]. Further, in high-resolution images (not shown), ~2–3 nm diameter pores are observed in the silica shells, and these voids are large enough to enclose a variety of small organic molecules. EDX analysis of the CaF_2_:Tb-MS NPs provides the estimate that these structures are ~61% silica and 36% CaF_2_:Tb by mass.

While mesoporous silica coatings on NPs are common in literature, applying a silica coating to a new NP system presents optimization challenges, including avoiding the aggregation of multiple NPs within single shells and self-nucleation of pure mesoporous silica NPs. While optimization is beyond the scope of this proof of principle synthesis and functionality assessment, we observed that the CaF_2_:TEOS ratio was crucial to control in order to reproducibly coat the CaF_2_:Tb NPs while maintaining a significant mass% of CaF_2_:Tb, which kept the formulation observably luminescent. Of the various ratios tested, 300 µL of TEOS to 54 mg of CaF_2_ in a 110 mL water/ethanol system was found to be optimal for minimizing particle aggregation related to surface charge. Lower concentrations of TEOS resulted in CaF_2_ without silica coatings, whereas higher concentrations of TEOS led to NP size increases due to coating thicknesses that would be prohibitive to cellular uptake or use as an RL agent.

Following silica coating, the CaF_2_:Tb-MS NPs were covalently functionalized with polyethylene glycol (PEG) using a PEG-silane precursor and conventional aqueous surface silanization methods. In general, PEG coatings are known to provide excellent water dispersability for NPs, while also lowering their zeta potential. The latter is thought to be critical in preventing NP opsonization, which is the electrostatic aggregation of proteins in biological systems that signals to immune cells that an object should be eliminated. This PEG coating makes the NPs in this work more applicable to the in vitro assays used to determine their XRT enhancement capability, however, intravenous applications would require additional surface modification. Delivery of NPs to tumors through passive accumulation results in low concentrations of NPs in the tumor tissues [41]. To increase the likelihood of the NP accumulation in a specific type of lesion, ligands would have to be attached to the surface such that the NPs were likely to bind to the target tissue’s cell membranes. For in vitro experiments, PEG alone was used to decrease the zeta potential of the NPs and prevent them from damaging cell membranes upon contact. As a dense layer of PEG is thought to be more effective in the prevention of NP opsonization, we employed basic conditions in order to promote complete and rapid hydrolysis of the PEG-silane precursor on to the CaF_2_:Tb-MS NP surfaces [42,43,44].

Briefly, the CaF_2_:Ln-MS-PEG NPs were synthesized by dispersing the CaF_2_:Ln-MS NPs in an aqueous solution containing a catalytic amount of aqueous base, then adding an excess of PEG-silane and stirring at room temperature. Compared to other combinations of weaker bases and anhydrous ethanol, ammonium hydroxide in water was found to result in attaching the largest amount of organic mass to the silica surface (Table S2). An analysis of the conditions of PEG-silane attachment is provided in the Supporting Information. Following PEGylation, the resulting CaF_2_:Ln MS-PEG NPs were collected via centrifugation and cured overnight at 60 °C. It has been shown that the addition of a curing step can aid in the attachment of weakly bound silanes to either the silica surface or to each other by dehydrating the material, thus encouraging dehydration condensation of the silanol groups on the silane and silica surface [45]. TEM evaluation indicated no significant morphological changes in the NPs following the covalent surface grafting with PEG-silane (Figure 3B). Additionally, DLS analysis revealed that CaF_2_:Tb-MS-PEG NPs dispersed in millipure water have a mean hydrodynamic diameter of 299 ± 2 nm, PDI of 0.372 ± 0.016, and a zeta potential of −32.2 ± 0.473 mV. The hydrodynamic diameter is greater than those observed by TEM imaging, suggesting that the NPs attract a significant layer of water around them, likely due to the hydrophilicity of PEG [46,47]. The charge at the surface of the NP is still partially negative, most likely left over from the partial protonation of remaining silanol groups on the silica surface [48].

The success of surface modification steps was validated by FT-IR spectroscopy, which was used to confirm the chemical identities of surface species for isolated NP products (Figure 3C). Prior to the addition of the mesoporous silica coating, the most prominent absorbances in the CaF_2_:Tb spectrum result from COO– stretching vibrations, which can be attributed to the citrate used as a stabilizer during synthesis [49]. After the addition of the silica coating, the Si–O–Si stretch becomes prominent at 1050 cm^−1^ (Figure 3C). Lastly, PEGylation of the CaF_2_:Tb-MS NPs is supported by the presence of new alkyl C–H stretches at 2900 cm^−1^, which can be attributed to the sp^3^ C–H bonds of the ethyl groups in PEG. Taken together, these findings confirm that the CaF_2_:Tb-MS-PEG NPs were successfully produced, and their radiological and biological properties are further examined in the following sections.

### 3.4. Radioluminescent Properties of CaF_2_:Ln Nanoparticles (NPs)

Photoluminescent CaF_2_:Ln NPs have been studied by several groups in the fields of biomedical diagnostics and therapeutics [20,21,22,23], as CaF_2_:Ln NPs can act as a source of visible-range photons, similar to other formulations of NP which enable imaging within biological tissues [15,16]. The same emitted photons can also photocatalyze specific chemical reactions, producing toxic species to kill local cells in a process commonly known as photodynamic therapy (PDT) [50,51,52]. One of the most prominent applications of CaF_2_:Ln NPs is in two-photon near-IR to visible energy conversion for deep tissue diagnostic and therapeutic applications. However, X-rays have greater biological tissue penetration depth as compared to near IR radiation, and consequently, interest in radioluminescence (RL) applications of CaF_2_ optical matrices has been stimulated [53].

While bulk CaF_2_ and Ln-doped CaF_2_ have been widely explored in RL applications, the RL characterization of doped CaF_2_ nanomaterials is less common. The intrinsic RL properties of undoped CaF_2_ NPs have been explored by Bezzera et al., who observed emission attributed to self-trapped excitons at 293 nm and from the F-centers at 428 nm [28]. Comparatively, Jacobsohn et al. investigated CaF_2_:Eu NPs, where irradiation with a 40 kV X-ray source resulted in emission intensity at 420 nm, indicative of the presence of Eu^2+^, as well as the expected Eu^3+^ emission transitions between 550–700 nm [54]. These studies illustrate that the RL properties of CaF_2_ and CaF_2_:Ln observed on the bulk scale are preserved in the nanoscale materials, which promoted our interest in exploring the potential of RL CaF_2_:Ln NPs in biomedical X-ray imaging and therapeutic applications.

**Figure 4:**
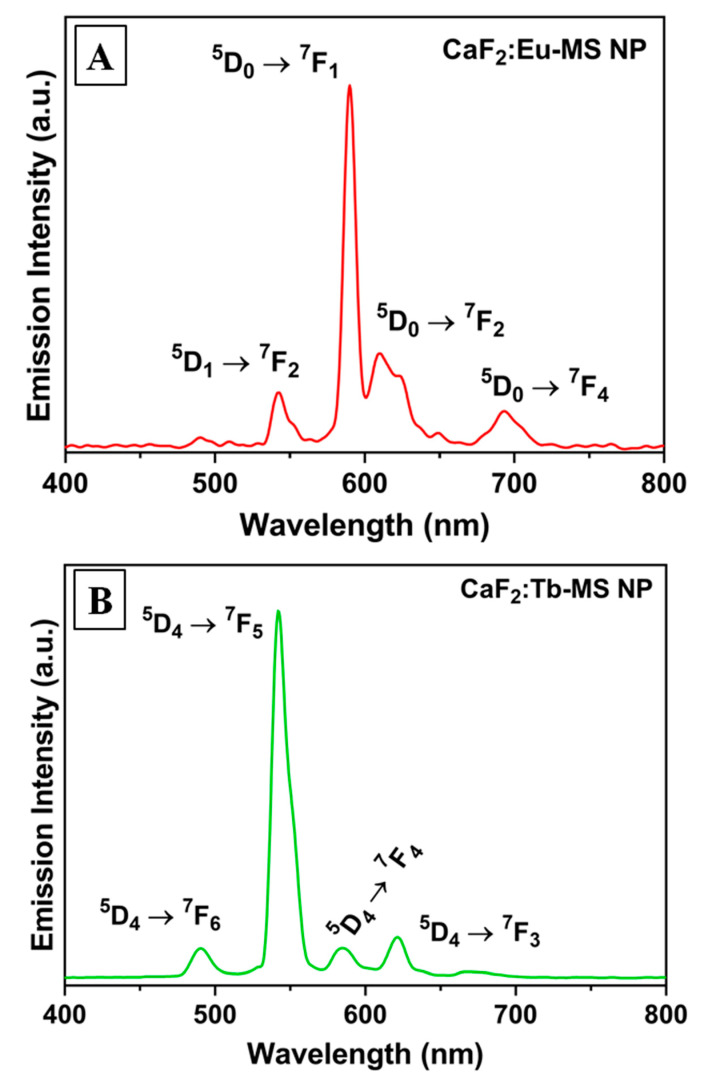
Radioluminescence emission spectra of (**A**) CaF_2_:Eu-MS NPs and (**B**) CaF_2_:Tb-MS NPs.

To assess radioluminescence, dry powders of CaF_2_, CaF_2_:Tb, and CaF_2_:Eu were irradiated at 130 kVp using a X-ray irradiator and visible light emission in the 400–800 nm range was monitored using a fiber-optic spectrophotometer. Each type of NP was assessed with and without the mesoporous silica shell. Expectantly, the undoped CaF_2_-MS NPs did not produce visible radioluminescence under these conditions (Figure S2), as the self-trapped exciton transition of CaF_2_ emits at too short of a wavelength to be observed with this RL measurement apparatus. In comparison, the lanthanide-doped CaF_2_-MS NPs displayed bright radioluminescence upon X-ray excitation (Figure 4). The CaF_2_:Eu-MS NPs exhibited distinct Eu emission peaks at 540 nm (^5^D_1_ → ^7^F_2_), 590 nm (^5^D_0_ → ^7^F_1_), 610 nm (^5^D_0_ → ^7^F_2_), and 693 nm (^5^D_0_ → ^7^F_4_), with the ^5^D_0_ → ^7^F_2_ emission at 590 nm being most intense. Comparatively, The CaF_2_:Tb-MS NPs displayed characteristic emission peaks at 490 nm (^5^D_4_ → ^7^F_6_), 542 nm (^5^D_4_ → ^7^F_5_), 586 nm (^5^D_4_ → ^7^F_4_), and 621 nm (^5^D_4_ → ^7^F_3_), of which the ^5^D_4_ → ^7^F_5_ transition at 542 nm displays the highest emission intensity. We compared the RL of these samples to the as-synthesized CaF_2_:Ln NPs, without the MS shell, and did not observe any significant spectral differences in the case of Ln = Tb (Figure S2). However, in the case of Ln = Eu, the MS shell addition increased the relative intensity of the ^5^D_1_ → ^7^F_2_ emission, which is perhaps attributable to a change in coordination environment of surface Ln ions (Figure S2) [55,56]. The relative emission intensities are known to be informative as to the coordination of the Eu dopant ions within the fluorite crystal structure and the dominance of the ^5^D_1_ → ^7^F_2_ transition at 591 nm implies that the dopants are replacing Ca^2+^ in the lattice [18,19]. Overall, these findings demonstrated the X-ray activation of lanthanide ion luminescence in the CaF_2_:Ln-MS NPs, and we further assessed the CaF_2_:Tb-MS NPs in cyotoxicology experiments, both in the absence and presence of incident X-radiation.

### 3.5. Cytotoxicity Assessment of CaF_2_:Tb NPs

**Figure 5:**
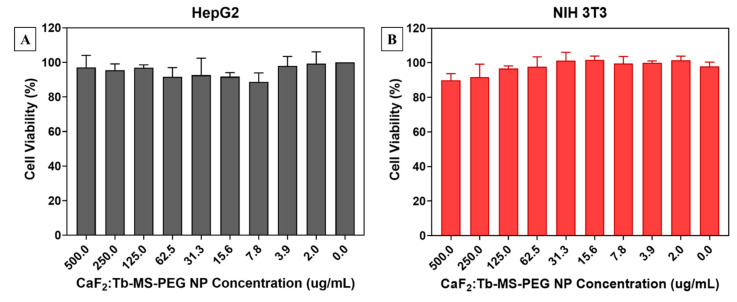
Cell viability assays of (**A**) HepG2 and (**B**) NIH 3T3 cell lines after incubation with various concentrations of CaF_2_:Tb-MS-PEG NPs for 24 h.

Following surface modification to prepare water-dispersible CaF_2_:Tb-MS-PEG NPs, cytotoxicity towards murine fibroblast (3T3 NIH) and human liver carcinoma (HepG2) was assessed. Here, cell viability was determined using an alamarBlue spectrophotometric assay, 24 h following exposure of the cells to the NPs at varying concentrations. As seen in Figure 5, CaF_2_:Tb-MS-PEG NPs displayed minimal cytotoxicity towards either cell line, at concentrations ranging from 2–500 µg/mL. The CaF_2_:Tb-MS-PEG NPs met our established threshold (>80% viability) for cytocompatibility in the absence of X-radiation, and the overall low in vitro toxicity renders them promising for in vivo biological applications.

### 3.6. Assessment of CaF_2_:Tb-MS-PEG NPs for X-ray Radiotherapy Enhancement

In cancer-based applications, CaF_2_-based biomaterials have been widely investigated as nanocarriers for therapeutic applications such as gene therapy, chemotherapy, photothermal therapy, and photodynamic therapy [57]. However, the reliance of some of these applications on excitation by near-IR photons introduces limitations due to poor tissue penetration by the incident light, which motivates further study into excitation using X-ray photons that have greater penetration depth [53]. A promising application that uses X-rays to excite nanomaterials is XRT enhancement, which has the goal of increasing local radiation damage in the vicinity of cancer-targeted NPs [2,58]. To date, however, only one other study has been published on the design of CaF_2_ NPs for X-ray activated therapy. In that work, Zahedifar et al. studied the use of RL CaF_2_:Tm NPs to excite the photosensitizer PPIX during X-ray irradiation. The results of this study showed good overlap between the RL emission of the NPs with the absorption band of PPIX, and indirect measurements of singlet oxygen generation suggest that the CaF_2_:Tm NP/PPIX system could be usefully applied under XRT conditions to enhance ROS generation [59]. Building on the progress of these studies, the following sets of experiments provide further evidence of CaF_2_-based NPs in the enhancement of cellular damage during XRT in vitro. As opposed to NP formulations which rely on singlet oxygen generation via a photosensitizer, this platform relies only on the conversion of X-ray photons to lower energy photons, enabled by the dense, crystalline NP.

**Figure 6:**
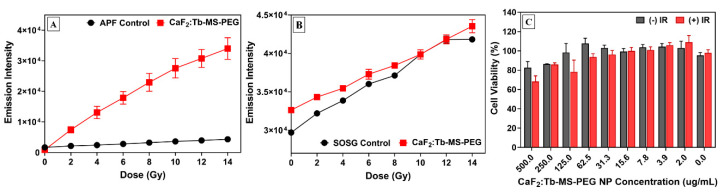
Assessment of CaF_2_:Tb-MS-PEG NPs for XRT. Fluorescent sensor XRT enhancement assays as a function of radiation dose for (**A**) hydroxyl radical using detection of aminophenyl fluorescein (APF) fluorescence intensity and (**B**) singlet oxygen using detection of Singlet Oxygen Sensor Green (SOSG) fluorescence intensity, with and without CaF_2_:Tb-MS-PEG NPs (125 μg/mL). In vitro cell viability assay (**C**) evaluating synergetic effects of XRT (4 Gy) and CaF_2_:Tb-MS-PEG NPs (0 to 500 μg/mL) on CT26 colorectal cancer cells; (+)-IR = with irradiation, (-)-IR = without irradiation.

Here, the ability of the CaF_2_:Tb-MS-PEG NPs to enhance ROS generation was spectroscopically assessed using two selective, molecular sensors, which allow quantification of hydroxyl radical and singlet oxygen production as a function of the applied X-ray radiation dose; control samples of water without NPs were measured under identical conditions (Figure 6A,B). Following interaction with the specific reactive species, the corresponding molecular sensors (Aminophenyl fluorescein (APF) for OH· and Singlet Oxygen Sensor Green (SOSG) for ^1^O_2_) become highly fluorescent, and the fluorescence intensity is known to increase in response to an increase in the concentration of the ROS analyte [60,61].

In these solution fluorescence spectroscopy experiments, we observed that upon X-ray irradiation, the CaF_2_:Tb-MS-PEG NPs greatly enhanced the production of hydroxyl radical as compared to the control solution. For the CaF_2_:Tb-MS-PEG NPs, the fluorescence intensity linearly increased as the radiation dose increased (Figure 6A). The observed increase in hydroxyl formation in the presence of the CaF_2_:Tb-MS-PEG NPs with increasing radiation dose is likely due to direct absorption of the incident energy by the CaF_2_:Tb NP cores followed by energy deposition via X-ray scattering; some of these scattered photons would be expected to result in downstream OH· production. However, it is possible that the incorporation of the silica layer in the CaF_2_:Tb-MS-PEG NPs may also be contributing to the observed increase in hydroxyl radical formation under XRT conditions, which is supported by prior work [62].

Comparatively, the incorporation of CaF_2_:Tb-MS-PEG NPs did not result in any significant increase in singlet oxygen generation as compared to the control solution (Figure 6B). These findings are unsurprising given that the excitation of dissolved O_2_ is typically achieved through a photosensitizer-mediated mechanism, and no purposeful photosensitizer is included in the present NP system. However, it should be noted that while SOSG is commonly used to determine singlet oxygen formation in PDT therapy using a UV-light source, it may not be a suitable probe for the detection of singlet oxygen in the presence of higher energy radiation. A recent report by Liu et al. found that following 320 kVp radiation, SOSG solutions demonstrated increasing fluorescence intensity with increasing radiation dose [63], which was found to be independent of singlet oxygen formation. These results suggest that the fluorescent probe is directly activated by the incident radiation exposure, which correlates well with our observation that the fluorescence intensity of the control solution increases somewhat linearly with the incident radiation dose and that the response is largely the same with or without NPs present (Figure 6B). Overall, the fluorescent probe XRT assays support the ability of the CaF_2_:Tb-MS-PEG NPs to enhance hydroxyl radical formation using incident X-rays, demonstrating them as potential radiosensitizing agents.

After spectroscopic evaluation of the CaF_2_:Tb-MS-PEG NPs for enhancing ROS generation under X-ray irradiation, cell viability studies were performed to assess their potential to act synergistically with XRT in vitro. Here, CaF_2_:Tb-MS-PEG NPs were incubated with murine CT26 colorectal cells at various concentrations (0 to 500 μg/mL) for 24 h, followed by irradiation of the experimental cell samples (+-IR) with a 4 Gy radiation dose; the viability of experimental cell samples was compared against un-irradiated control cells ((-)-IR). Twenty-four hours after irradiation, cell viability was assessed using the alamarBlue assay. This assay measures the metabolic activity of a cell population, which can be correlated to the fraction of living cells. As shown in Figure 6C, there is some correlation between decreased cell viability and CaF_2_:Tb-MS-PEG NP concentration, particularly at higher concentrations (500–31.3 µg/mL). In the absence of CaF_2_:Tb-MS-PEG NPs, exposure to ionizing radiation resulted in 98% cell viability over the given time frame, demonstrating good cytocompatibility of the particles in the absence of X-radiation. Comparatively, exposure and incubation with 125 and 500 µg/mL of CaF_2_:Tb-MS-PEG NPs resulted in decreasing cell viability at 78% and 68%, respectively. Some conditions led to mean % viabilities above 100%, which may be attributed to NPs interacting with cells and possibly increasing their metabolic or proliferative rate above that of the control population.

An important aspect of combination therapies is maximizing the anticancer effects while minimizing toxicity to healthy tissues. Given the high biocompatibility in the absence of radiation, and preliminary studies showing dose-dependent enhancement of cell kill with radiation, the CaF_2_:Tb-MS-PEG NPs demonstrate promising characteristic for use as an NP radiotherapeutic agent. Our further work will optimize the structural (e.g., size), spectral (e.g., absorption/emission spectral overlaps), and surface chemical features (e.g., targeting ligand display) of CaF_2_:Ln NPs to further increase the radiotherapeutic efficacy of this NP system.

## 4. Conclusions

Overall, this study has presented the synthesis, characterization, and evaluation of CaF_2_- core mesoporous silica-shell NPs as X-ray activated materials for radioluminescence and radiotherapy enhancement applications. The CaF_2_ and Ln-doped CaF_2_ NPs were synthesized using an aqueous coprecipitation method. Hydrothermal annealing was then used to fuse small crystallites and improve the luminescence intensity of the Ln-doped materials. Surface modifications through mesoporous silica coating and PEGylation post-core-synthesis resulted in water-stable particles that displayed excellent cytocompatibility. Characterization of the radioluminescence properties of the Ln doped CaF_2_ NPs demonstrated their potential for RL-based applications. Additionally, their potential efficacy as a radiosensitizing material was demonstrated in vitro with enhanced cell kill, serving as an initial proof of concept demonstration. This is supported by the observed ability of the CaF_2_ NPs to enhance hydroxyl radical formation following X-ray irradiation. Moving forward, overall material efficacy may be improved by the addition of surface ligands to the NPs for targeted therapy. Additionally, the luminescence of the CaF_2_ core could potentially be used to enable X-ray excited photodynamic therapy, which would have the advantages of the current formulation while providing additional routes for radical production.

## Figures and Tables

**Table 1:** Metal chloride masses added to flasks to attain certain doping amounts.

Synthesis Target (Dopants in Atomic %)	Calcium Chloride Mass (mg)	Doping Metal Chloride Mass (mg)
Undoped CaF_2_ NPs	388	None
CaF_2_:Tb 18% NPs	350	93 TbCl_3_·6H_2_O
CaF_2_:Eu 18% NPs	350	90 EuCl_3_·6H_2_O

## Supplementary Materials

The following are available online at https://www.mdpi.com/2079-4991/10/8/1447/s1, Table S1: Diameter data collected by measuring NPs with different doping qualities. Figure S1: EDX spectrum of CaF_2_:Tb NPs and EDX-derived % atomic compositions of CaF_2_ NPs. Table S2: XPS-derived Atomic % concentrations measured from the surface of differently treated silica NPs. Figure S2. Comparison of X-ray luminescence spectra of doped CaF_2_ NPs before and after coating with mesoporous silica. Relative emission peak intensities appear unaffected by the coating, and luminescence was not observed in the absence of dopants.
