# Supplementary material for: Synthesis of Radioluminescent CaF2:Ln Core, Mesoporous Silica Shell Nanoparticles for Use in X-ray Based Theranostics
Source: Nanomaterials (Basel). 2020 Jul 24;10(8):1447. doi: 10.3390/nano10081447 (PMC7466269; doi:10.3390/nano10081447)
Supplement: Supplementary file 1 [file nanomaterials-10-01447-s001.pdf]

nanomaterials

Supplementary Information

Synthesis of Radioluminescent CaF_2:Ln Core, Mesoporous Silica Shell Nanoparticles for use in X-ray Based Theranostics

Hayden Winter [1], Megan J. Neufeld [2], Lydia Makotamo [1], Conroy Sun [2,3,*], and Andrea M. Goforth [1,*]

[1] Department of Chemistry, Portland State University, 1719 SW 10th Ave., Portland, OR 97201, United States; hwinter@pdx.edu(H.W.); lym2@pdx.edu (L.M.)
[2] Department of Pharmaceutical Sciences, College of Pharmacy, Oregon State University, Portland, Oregon 97201, United States; neufeldm@oregonstate.edu
[3] Department of Radiation Medicine, 3181 S. W. Sam Jackson Park Rd, Oregon Health & Science University, Portland, Oregon 97239, United States
* Correspondence: amgofort@pdx.edu (A.M.G.); sunc@ohsu.edu (C.S.)

Table S1. Diameter data collected by measuring NPs with different doping qualities.

NP Sample	Mean Diameter (nm)	Standard Deviation (± nm)
CaF_2	13.6	3.5
CaF_2:Tb	13.4	4.2
CaF_2:Eu	12.5	3.3

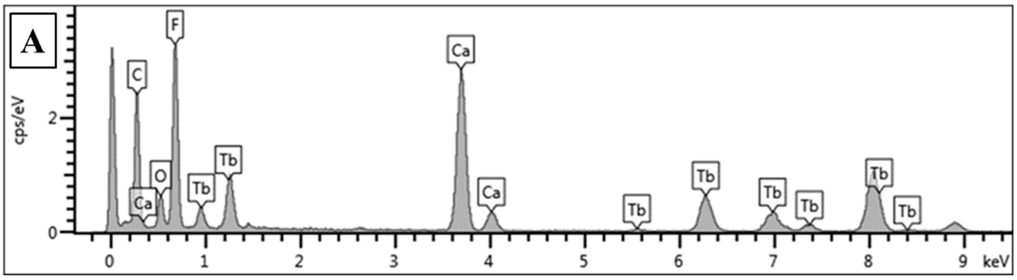

B

Sample	Ca Atomic %	F Atomic %	Dopant Atomic %	Doping %
CaF_2	30.54	64.38	--	0
CaF_2:Tb	27.88	60.62	(Tb) 6.20	18.2
CaF_2:Eu	29.52	58.72	(Eu) 5.56	18.8

Figure S1. (**A**) EDX spectrum of CaF_2:Tb NPs and (**B**) EDX-derived % atomic compositions of CaF_2 NPs.

Table S2. XPS-derived Atomic % concentrations measured from the surface of differently treated silica NPs.

Conditions	Atomic % Si	Atomic % C	Ratio
Untreated Silica	17.5	37.3	1:2.1
Water/Triethylamine	8.5	31.7	1:3.9

Water/NH_4OH	9.0	45.7	1:5.1
Ethanol/ Triethylamine	11.8	38.1	1:3.2

Analysis of Table S2: XPS experiments were performed to confirm that aqueous conditions with a strong base attached the most PEG to the surface out of all of the conditions tested. CaF_2 is not compatible with the VersaProbe XPS available due to the XPS' LaB_6 electron beam generator. Commercially available silica nanoparticles (Ludox) were used as a stand-in for the silica surfaces. Four conditions were tested, using a combination of anhydrous ethanol or water as a reaction solvent, and the strong base NH_4OH as a catalyst or the weak base triethylamine. While a combination of weak bases and anhydrous solvents is common in silanization literature, groups who do these reactions are often striving to achieve uniform monolayers of silane on their surfaces, whereas our goal was to maximize PEG attachment. The optimized parameter was the ratio of silicon to carbon that could be observed by the XPS, with a higher ratio of carbon being indicative of a thicker or denser coating of PEG. The results are summarized in Table 3. These values must be examined in relation to each other, as the exact atomic % will be variable with the penetration depth of the X-rays into the material, which will change with PEG coating thickness. The highest atomic ratio observed was in the sample prepared under the combined NH_4OH and water parameters, which encouraged the most rapid hydrolysis of the PEG silane.

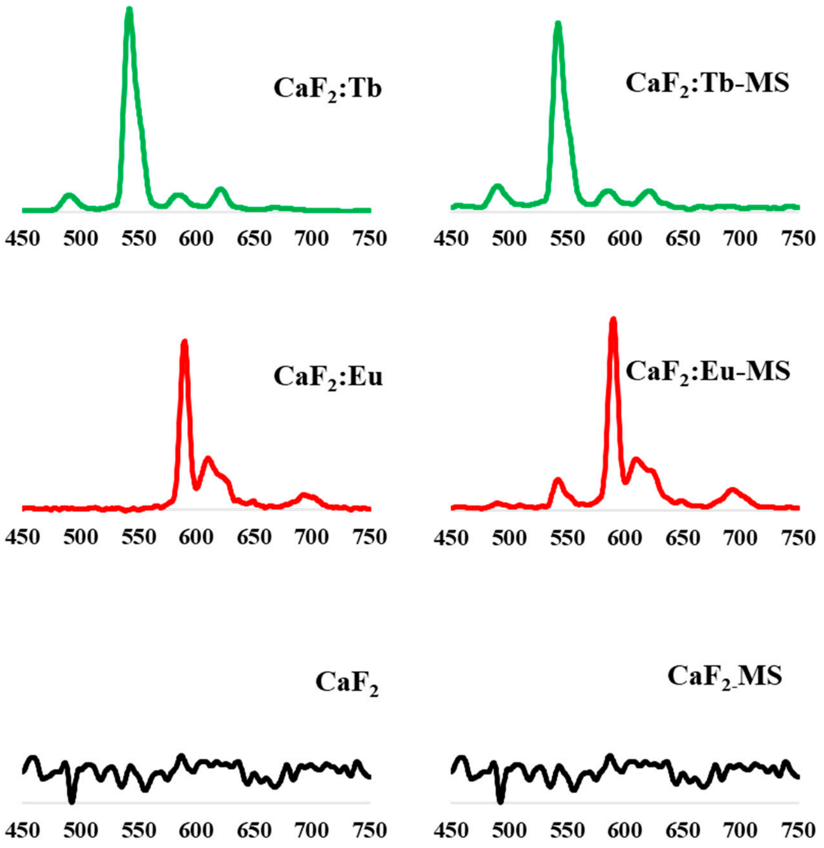

Figure S2. Comparison of X-ray luminescence spectra of doped CaF2 NPs before and after coating with mesoporous silica. Relative emission peak intensities appear unaffected by the coating, and luminescence was not observed in the absence of dopants.

© 2020 by the authors. Licensee MDPI, Basel, Switzerland. This article is an open access article distributed under the terms and conditions of the Creative Commons Attribution (CC BY) license (http://creativecommons.org/licenses/by/4.0/).
